# Consistency of magnetoencephalographic functional connectivity and network reconstruction using a template versus native MRI for co‐registration

**DOI:** 10.1002/hbm.23827

**Published:** 2017-10-08

**Authors:** Linda Douw, Dagmar Nieboer, Cornelis J. Stam, Prejaas Tewarie, Arjan Hillebrand

**Affiliations:** ^1^ Department of Anatomy and Neurosciences VU University Medical Center Amsterdam The Netherlands; ^2^ Department of Radiology Athinoula A. Martinos Center for Biomedical Imaging/Massachusetts General Hospital Boston Massachusetts; ^3^ Department of Methodology and Applied Biostatistics, Faculty of Science VU University Amsterdam Amsterdam The Netherlands; ^4^ Department of Clinical Neurophysiology and Magnetoencephalography Center VU University Medical Center Amsterdam The Netherlands; ^5^ Sir Peter Mansfield Imaging Centre, School of Physics and Astronomy University of Nottingham Nottingham United Kingdom

**Keywords:** magnetoencephalography, co‐registration, beamforming, MNI template, MRI, functional connectivity, network analyses, consistency

## Abstract

**Introduction:**

Studies using functional connectivity and network analyses based on magnetoencephalography (MEG) with source localization are rapidly emerging in neuroscientific literature. However, these analyses currently depend on the availability of costly and sometimes burdensome individual MR scans for co‐registration. We evaluated the consistency of these measures when using a template MRI, instead of native MRI, for the analysis of functional connectivity and network topology.

**Methods:**

Seventeen healthy participants underwent resting‐state eyes‐closed MEG and anatomical MRI. These data were projected into source space using an atlas‐based peak voxel and a centroid beamforming approach either using (1) participants’ native MRIs or (2) the Montreal Neurological Institute's template. For both methods, time series were reconstructed from 78 cortical atlas regions. Relative power was determined in six classical frequency bands per region and globally averaged. Functional connectivity (phase lag index) between each pair of regions was calculated. The adjacency matrices were then used to reconstruct functional networks, of which regional and global metrics were determined. Intraclass correlation coefficients were calculated and Bland–Altman plots were made to quantify the consistency and potential bias of the use of template versus native MRI.

**Results:**

Co‐registration with the template yielded largely consistent relative power, connectivity, and network estimates compared to native MRI.

**Discussion:**

These findings indicate that there is no (systematic) bias or inconsistency between template and native MRI co‐registration of MEG. They open up possibilities for retrospective and prospective analyses to MEG datasets in the general population that have no native MRIs available. *Hum Brain Mapp, 2017*. © **2017 The Authors Human Brain Mapping Published by Wiley Periodicals, Inc.** *Hum Brain Mapp 39:104–119, 2018*. © **2017 Wiley Periodicals, Inc.**

## INTRODUCTION

Magnetoencephalography (MEG) is a technique often used to investigate functional connectivity and network topology of the brain [Stam, 2014]. MEG records the magnetic fields that are induced by neuronal currents, providing information about normal and pathological processes in the brain with high temporal and good spatial resolution [Buzsaki, 2006; Schnitzler and Gross, 2005]. It also allows for the accurate estimation of statistical interdependencies between time series of neural activity recorded from different brain regions, termed functional connectivity [Aertsen et al., 1989; Friston, 1994].

Many earlier MEG functional connectivity studies, including our own, have employed a “signal‐space” approach, meaning that signals measured from outside the scalp are directly correlated and interpreted [Douw et al., 2014; Fernández et al., 2006; Nieboer et al., 2016; Olde Dubbelink et al., 2008]. However, this type of analysis does not allow for interpretation of estimated activity or connectivity patterns in an anatomical context; a solution to the inverse problem is required for this. Beamforming [Hillebrand et al., 2005] is one of the popular source reconstruction approaches [Baillet et al., 2001] that yield plausible and verifiable results when combined with an anatomical image of the individual's skull and brain by, for example, T1‐weighted magnetic resonance imaging (MRI) [Barnes et al., 2006; Hillebrand and Barnes, 2005]. The relevance of this technique for atlas‐based connectivity and functional network studies has amply been shown [Hillebrand et al., 2012, 2016].

However, individual anatomical MRIs may not be available in certain (retrospective) datasets and the time and effort of obtaining an anatomical MRI may put an extra burden on patients. Using an MRI template instead of a native MR scan for source localization could minimize these cons and render the use of source‐localized MEG data feasible in such cases. At the same time, individual anatomy, although the current standard for analysis in most source‐space studies, may not necessarily yield superior results: the spatial resolution of MEG may generally allow for atlas‐based connectivity and network analytical studies, be it using template or native anatomical imaging for co‐registration.

Several studies have already employed a template‐based method for source localization in the absence of native MRIs. For instance, López et al. [2014] have used a template generated from MRIs of healthy controls for source reconstruction to perform connectivity analysis. Holliday et al. [2003] selected the MRI with closest matching external head shape from a database, and used it as a substitute for the subject's own MRI. Another research group derived geometrical information from the volume conductor models that were constructed during MEG analysis, that is, information based on data from an external digitizer, in combination with a template MRI to source‐localize magnetic activity [Steinstraeter et al., 2009]. A digitizing pen (Polhemus) is often used to project the *X*, *Y*, and *Z* coordinates of the coil locations onto the cortical surface following the co‐registration with the corresponding MRI. Similarly, it has been shown that external landmarks or the external head shape, again based on digitized scalp surfaces, can be used in combination with a template head‐shape to align functional images with a structural template [Beg et al., 2009]. However, an analysis of possible inconsistencies, particularly with respect to connectivity and network analyses, resulting from the use of a template MRI as compared to a native MRI has not been performed. It may be that highly consistent results between these approaches can be obtained at the level of individual time series and relative power, but that increases in inconsistency arise when investigating connectivity and network patterns. This study is therefore an addition to the previous studies that already used some sort of template warping as it specifically addresses whether (possibly nonlinear) inconsistencies feed into different higher order outcome measures obtained with the template versus native MRI approach.

Here, we evaluated the consistency and potential bias of estimates of spectral power, functional connectivity, and network topology in a group of healthy subjects, using either the MNI template or their native anatomical MR scans for MEG source reconstruction.

## METHODS

### Participants

A previously described dataset consisting of data collected in 17 healthy participants aged 30–59 years (39.8 ± 9.8) was used. These participants did not suffer from neurological or psychiatric diseases and did not use any drugs or medication. The study was approved by the Medical Ethical Committee of the VU University Medical Center (Amsterdam, The Netherlands), and all participants gave written informed consent before participation. Data from these healthy volunteers have been used as a control group in previous case–control studies from our group [Tewarie et al., 2014a; Tewarie et al., 2015].

### Magnetoencephalography

Magnetic fields were recorded for five minutes while subjects were in supine position inside a magnetically shielded room (Vacuumschmelze GmbH, Hanau, Germany), during eyes‐closed resting state. We used a 151‐channel whole‐head MEG system (CTF Systems Inc., Port Coquitlam, BC, Canada). A third‐order software gradient [Vrba et al., 1999] was used with a recording passband of 0–150 Hz and a sampling frequency of 625 Hz. At the beginning and end of each recording, the head position relative to the coordinate system of the helmet was recorded by passing small alternating currents through three head position coils attached to the left and right preauricular points and the nasion. Head movements of up to 0.5 cm were allowed during recording. For each subject, 45 epochs of 4,096 samples (6.554 s) were recorded. From these, all artifact‐free epochs, that is, those not containing system related artifacts, physiological artifacts, metal artifacts and environmental noise, were selected by one of the authors [PT]. On average 35 (range 21–43) epochs per subject were used for further analysis. These epochs were band‐pass filtered into the six classical frequency bands using a discrete Fast Fourier Transform: delta (0.5–4 Hz), theta (4–8 Hz), lower alpha (8–10 Hz), upper alpha (10–13 Hz), beta (13–30 Hz), and gamma (30–48 Hz).

### Co‐Registration With Native and Template MRI

**Figure 1:**
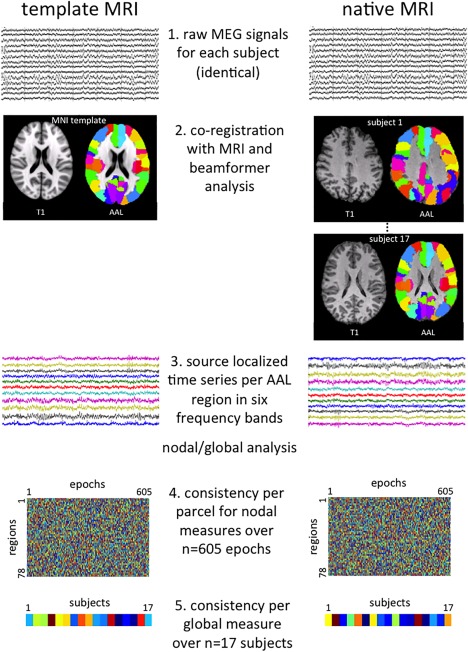
Schematic of the analysis pipeline. Important steps in the data analysis pipeline are depicted for both the analysis with the template MRI (left) and the native MRI (right). Step 1 indicates the raw (sensor level) magnetoencephalography (MEG) recordings, which are identical for both methods. In Step 2, co‐registration of the MEG data takes place with either the template MRI (left) or subjects’ native MRIs (right), here depicted with the AAL atlas overlaid. Step 3 involves extraction of time series per AAL region for each frequency band using beamforming, based on which power, connectivity, and network characteristics are calculated (regional/global analysis). In Step 4, we illustrate the final data structure for one regional measure (power, phase lag index, or minimum spanning tree degree): a matrix of 78 regions by 605 epochs (= 17 subjects × on average 35 (range 21–43) epochs per subject). Consistency between each row in these matrices was then tested, yielding an intraclass correlation coefficient (ICC) per AAL region. Step 5 shows the final data structure per global measure: a vector of values for 17 subjects, which was calculated for average power, average phase lag index, and the global network measures. The ICC of these two vectors was computed for each global measure. [Color figure can be viewed at http://wileyonlinelibrary.com]

The data analysis pipeline is schematically depicted in Figure 1. The current standard of analysis for this type of data is co‐registration with an individual's anatomical MRI. To this end, an MRI of the head was obtained at 3 T (GE SignaHDxt), with a 3D‐T1 weighted fast spoiled gradient‐echo (FSPGR, TR 7.8 ms, TE 3.0 ms, TI 450 ms, flip angle 12°, 0.9 × 0.9 × 1 mm voxel size). Vitamin E capsules were placed at aforementioned anatomical landmarks, that is, the preauricular points and the nasion, to guide co‐registration with the MEG data. Using these two corresponding sets of fiducial markers, the MEG and MRI coordinate systems were matched (i.e., the two sets of 3 points were aligned using a rigid body transformation (rotation and translation) [Fitzpatrick et al., 1998], using MRIViewer (version 5.0.2, CTF Systems Inc.)). The co‐registered MRI was subsequently segmented, and the outline of the scalp was used to compute a multisphere head model [Huang et al., 1999] for the calculation of the lead‐fields. Co‐registration with a template MRI instead of native imaging was investigated using the T1‐weighted MNI template with 1 mm resolution [Smith et al., 2004], which is available from the FSL software package (http://fsl.fmrib.ox.ac.uk/fsl/fslwiki/).

### Atlas‐Based Beamforming

The source reconstruction approach has been described in detail [Hillebrand et al., 2012]. In short, a co‐registered MRI (either template or native in MEG space) was spatially normalized to the template MRI. To label the voxels in a subject's normalized co‐registered MRI, the AAL atlas was used [Tzourio‐Mazoyer et al., 2002]. After inverse transformation to the patient's co‐registered MRI, the 78 cortical regions of interest (ROIs) were used for further analysis [Gong et al., 2009]. Furthermore, the neuronal activity for the labeled voxels in the 78 ROIs, using a grid‐size of 2 mm, was reconstructed using a beamformer approach known as Synthetic Aperture Magnetometry (SAM) [Robinson, 1999]. The neuronal activity at each voxel, a so called virtual electrode (VE), was reconstructed as the weighted sum of the recorded magnetic field B at a given time point as [van Veen et al., 1997]:

(1) $$\mathrm{VE}={\left(L^{T}C_{b}^{-1}L\right)}^{-1}L^{T}C_{b}^{-1}B=W^{T}B,$$

Here, T stands for transpose and W stands for the beamformer weights, which are determined by the lead fields (L) and the data covariance matrix (C_b_) (which is based on data from on average 229 s (range: 138–282s)).

### Peak Voxel and Centroid Approaches

To represent each ROI with a single time series, two different approaches were used. Firstly, the voxel with maximum pseudo‐*Z* value in each frequency band for each ROI was selected, as has been reported previously [Hillebrand et al., 2012; Robinson, 1999]. This resulted in 78 time series for each frequency band for further analysis. However, the choice of peak voxel might be influenced by outliers more strongly than other approaches. Therefore, additionally, we used the centroid as representative for each ROI, an approach that has also been used by our group before [Hillebrand et al., 2016]. The centroid is defined here as the voxel within the ROI that is nearest, in terms of Euclidean distance, to all other voxels in the ROI.

### Consistency of Virtual Electrode Locations

To scope the relationship between inconsistencies in the virtual electrode locations obtained with the two approaches and the consistency of band‐limited relative power, we determined the Euclidean distance between the location of the representative voxel for each ROI (either determined by peak voxel or centroid) as obtained with the template approach and the location as obtained when using the subject's own MRI.

### Relative Power

All following analyses were performed using BrainWave [CJS, version 0.9.133, available from http://home.kpn.nl/stam7883/brainwave.html]. We investigated band‐specific relative power in each of the six frequency bands by applying a Fast Fourier transform to the time series per region in the AAL atlas.

### Connectivity and Network Analysis

As a measure of functional connectivity, the phase lag index (PLI) was used [Stam et al., 2007], which calculates the asymmetry of the distribution of (instantaneous) phase differences between two time series. The asymmetry of the distribution of phase differences of two signals can be obtained from a time series of phase differences ΔΦ(*t_k_*), *k* = 1…*N* samples:

(2) $$\text{PLI}=|<\text{sign}\left[\text{sin}\left(\Delta \varphi \left(t_{k}\right)\right)\right]>|$$

The presence of a consistent, nonzero, phase lag between two time series reflects true interactions that are unaffected by the effects of volume conduction or field spread, also referred to as leakage in source space. Frequency‐specific connectivity between all region pairs was calculated for each epoch. A weighted adjacency matrix was then constructed, having the AAL regions as rows and columns and the connectivity values as entries. Global and regional network characteristics were determined using several previously described measures for each epoch and frequency band: the classical graph measures of weighted clustering coefficient and weighted path length [Bullmore and Sporns, 2009; Stam and Reijneveld, 2007] and modularity [Newman, 2006], and characteristics of the minimum spanning tree (MST) [Kruskal, 1956; Stam et al., 2014; Tewarie et al., 2015].

The clustering coefficient is defined as the probability that a node's neighbors are also connected to each other [Watts and Strogatz, 1998]. In a weighted network, clustering also takes into account the strength of each connection; in our case, the PLI value between each region pair [Stam et al., 2009].

The path length assesses the integration of the network, by computing the number of steps making up the shortest possible path between every node pair. In our weighted case, this path length incorporates the strength of the PLI in this shortest path length by using Dijkstra's Algorithm [Dijkstra, 1959]. The combination of high clustering and short average path length makes up the “small‐world” topology [Watts and Strogatz, 1998], which is thought to be optimal for information processing.

Modularity refers to the extent to which the network is organized into subsystems or modules. Newman's modularity algorithm was used to calculate the optimal modular division into strongly intraconnected but weakly interconnected modules for each adjacency matrix, in which each region received a single‐module allegiance [Newman, 2006]. This analysis yielded a global measure of modularity (also termed “Q”), and the number of modules resulting from the network decomposition. As the modularity algorithm depends on simulated annealing to obtain the optimal partition of the network, it yields slightly different results in every run, possibly increasing the noise level that may already be present when using different images for co‐registration. We therefore ran the modularity algorithm 100 times and averaged the values of Q into a single, stable measure of modularity within each subject.

The minimum spanning tree (MST) is a subnetwork of the original weighted network that connects all nodes without forming loops and has the minimum total weight of all possible spanning trees, where edge weight is defined as 1/PLI. The MST was constructed based on the weighted networks with Kruskal's algorithm [Kruskal, 1956], and is thought to deduce a bias‐free backbone of the original weighted network. We characterized the topology of the MSTs using the following measures: degree, which is the number of connections for each node in the tree; leaf fraction, referring to the fraction of nodes in the tree with a degree of one; and diameter, that is, the diameter is the longest distance between any two nodes of the tree. These network measures have recently been used in population and clinical studies [Stam et al., 2014].

### Statistical Analysis

Statistical analyses were performed using SPSS Statistics package version 20.0 (IBM, Armonk (NY), USA) and Matlab version r2012b (MathWorks, Natick (MA), USA). The consistency of spectral power, functional connectivity, and network measures between the two approaches, that is, using native MRI or the template, were investigated by calculating intraclass correlation coefficients (ICC) with a two‐way mixed model, ICC(3,1) [McGraw and Wong, 1996; Shrout and Fleiss, 1979]. In this two‐way mixed model, the raters or approaches (template and native MRI) are considered fixed effects, and each item (MEG measure for an epoch) is treated as a random effect. The ICC is commonly used to assess the consistency of quantitative measurements made by different “observers” as it takes the rater bias into account, as consistency not only requires high correlation but also requires small rater bias. In our case, the “observers” are the two different approaches.

The first step was to investigate whether the distance between the locations of the virtual electrodes as obtained with the template approach and the location as obtained when using the subject's own MRI was different between the peak voxel and centroid methods with an ANOVA and post‐hoc Student's *t* tests. Also, for the peak voxel method, the distance for each ROI was plotted against the ICC of the band‐limited relative power for each ROI to visualize the relationship between the inconsistencies in the virtual electrode locations and the ICCs. This was quantified using Pearson correlation

We then explored the consistency of the time series as extracted with the template and native MRI approach. To obtain an indication of this consistency, we report for each frequency band the ICC of the time series for those ROIs with the lowest and highest consistency of the peak voxel locations

Furthermore, the ICC values were calculated at two levels (Fig. 1): the first one relates to the assessment of consistency of the template versus native MRI approach per region for each epoch (*n* = 605) for the regional measures of power, PLI, and MST degree. Power and PLI are the basis for the following network analyses, and therefore we were interested in their consistency at the fine‐grained spatial (i.e., per region) and temporal (i.e., per individual epoch) levels.

Third, we were also interested in the consistency of template versus native MRI results with respect to subject‐level characteristics, as most studies make use of subject‐averaged values. We therefore averaged several global measures, such as average power, average PLI, clustering coefficient, path length, modularity, number of modules, MST leaf fraction, and MST diameter, over all epochs per subject (*n* = 17).

The ICC values were interpreted as follows: >0.80 very good, 0.61–0.80 good, 0.41–0.60 moderate, 0.21–0.40 fair, and <0.21 poor [Brennan and Silman, 1992]. To further scope consistency of template versus native MRI co‐registration of MEG, Bland–Altman plots were constructed. These plots are based on a visualization of the mean and standard deviation of the difference of the measurement by two approaches. It evaluates the agreement and possible bias between them [Altman and Bland, 1983]. The 95% limits of agreement, which are defined as the mean difference plus and minus 1.96 times the standard deviation of the difference, are added in the plot to judge how well the two approaches agree upon a certain value. This approach is the most commonly used approach to give more insight in the existence of any systematic difference between two different methods [Zaki et al., 2012].

## RESULTS

### Consistency of Virtual Electrode Locations

**Figure 2:**
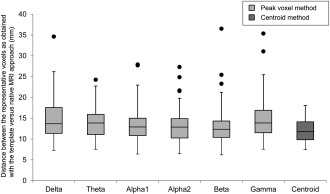
Distance between native and template‐based representative voxels. Box plots are shown for the distance between the 78 representative voxels as obtained with the template and native MRI approach. Frequency bands indicate those voxels obtained with the peak voxel method for the different bands (light grey) while the distance was equal for all frequency bands using the centroid method (dark grey).

The average distance between the virtual electrode locations as obtained with the template versus native MRI differed between the peak voxel and centroid method (ANOVA (*F*(6,539) = 4.2, *P* < 0.001), in which the centroid method showed a smaller average distance and less variation (Fig. 2), indicating that it is likely to give better results with regard to consistency of the other outcome measures (see also Table 1 versus Supporting Information, Appendix 1). To simplify this results section, but avoid any overestimation of the consistency between the template versus native MRI approach, we will proceed reporting only the supposedly less optimal peak voxel results throughout this article (see Supporting Information, Appendix 1 for results obtained with the centroid method).

**Table 1:** Global consistency between results obtained when using the template and native MRI approach (with the peak voxel method)

	Delta			Theta			Lower alpha		
	ICC	95% CI	Rating	ICC	95% CI	Rating	ICC	95% CI	Rating
Power	0.988	0.940–0.996	VG	0.994	0.983–0.998	VG	0.966	0.803–0.990	VG
PLI	0.932	0.817–0.975	VG	0.870	0.637–0.953	VG	0.918	0.644–0.975	VG
Cw	0.911	0.739–0.968	VG	0.733	0.297–0.901	G	0.906	0.529–0.972	VG
Lw	0.944	0.780–0.982	VG	0.849	0.420–0.951	VG	0.926	0.798–0.973	VG
Modularity	0.670	0.119–0.879	G	−0.325^a^	−0.209–0.448	NR	0.555	−0.160–0.835	M
Number of modules	0.594	−0.068–0.85	M	0.551	−0.113–0.83	M	0.744	−0.027–0.921	G
MST leaf fraction	0.754	0.134–0.920	G	0.689	−0.006–0.895	G	0.623	−0.066–0.868	G
MST diameter	0.828	0.536–0.937	VG	0.840	0.572–0.942	VG	0.327	−0.764–0.752	F

	Upper alpha			Beta			Gamma		
	ICC	95% CI	Rating	ICC	95% CI	Rating	ICC	95% CI	Rating
Power	0.969	0.497–0.993	VG	0.993	0.918–0.998	VG	0.903	0.285–0.974	VG
PLI	0.937	0.431–0.984	VG	0.895	0.711–0.962	VG	0.155	−0.043–0.524	P
Cw	0.917	0.196–0.980	VG	0.864	0.634–0.950	VG	0.179	−0.081–0.561	P
Lw	0.957	0.803–0.987	VG	0.905	0.743–0.965	VG	0.123	−0.022–0.459	P
Modularity	0.766	0.371–0.915	G	0.256	−0.698–0.708	F	0.560	−0.087–0.838	M
Number of modules	0.695	−0.062–0.901	G	0.670	0.145–0.877	G	0.320	−0.558–0.734	F
MST leaf fraction	0.717	−0.048–0.911	G	0.774	0.392–0.917	G	0.698	0.150–0.892	G
MST diameter	0.571	−0.179–0.848	M	0.577	−0.058–0.841	M	0.635	0.057–0.865	G

*N* = 17; df 16, ICC, intraclass correlation coefficient ; 95 CI, 95% confidence interval; VG = very good ICC >0.80; G = good ICC 0.61–0.80; M = moderate ICC 0.41–0.60; F = fair ICC 0.21–0.40; P = poor ICC <0.21.

a Not reliable.

### Consistency of the Time Series

For the ROIs with the lowest and highest consistency of the peak voxel locations, median ICCs and their classification can be seen in Table 2. These results show very good consistency of time series when spatial consistency was also high, while the consistency of time series was still very good in most frequency bands when spatial consistency was low. Only the theta and gamma band showed suboptimal ICCs of the time series for these ROIs with low spatial consistency.

**Table 2:** Consistency of time series extracted using the template versus native MRI approach

ROIs with lowest consistency in peak voxel location				ROIs with highest consistency in peak voxel location		
Frequency band	ROI	Median ICC	Rating	ROI	Median ICC	Rating
Delta	Occipital Inf. R	0.91	VG	Frontal Mid. Orb. L	0.90	VG
Theta	Heschl L	0.41	M	Occipital Inf. L	0.96	VG
Alpha1	Frontal Inf. Oper. R	0.90	VG	Cuneus L	0.93	VG
Alpha2	Lingual L	0.94	VG	Calcarine L	0.95	VG
Beta	Occipital Inf. R	0.96	VG	Frontal Mid. Orb. L	0.91	VG
Gamma	Frontal Mid. L	0.24	F	Calcarine R	0.91	VG

ROI, region of interest; R, right; L, left; ICC, intraclass correlation coefficient.

VG = very good ICC >0.80; G = good ICC 0.61–0.80; M = moderate ICC 0.41–0.60; F = fair ICC 0.21–0.40; P = poor ICC <0.21.

ICCs are reported for ROIs with the lowest and highest consistency of the peak voxel locations in each frequency band.

### The Impact of the Consistency of Virtual Electrode Locations on the ICC of Relative Power

**Figure 3:**
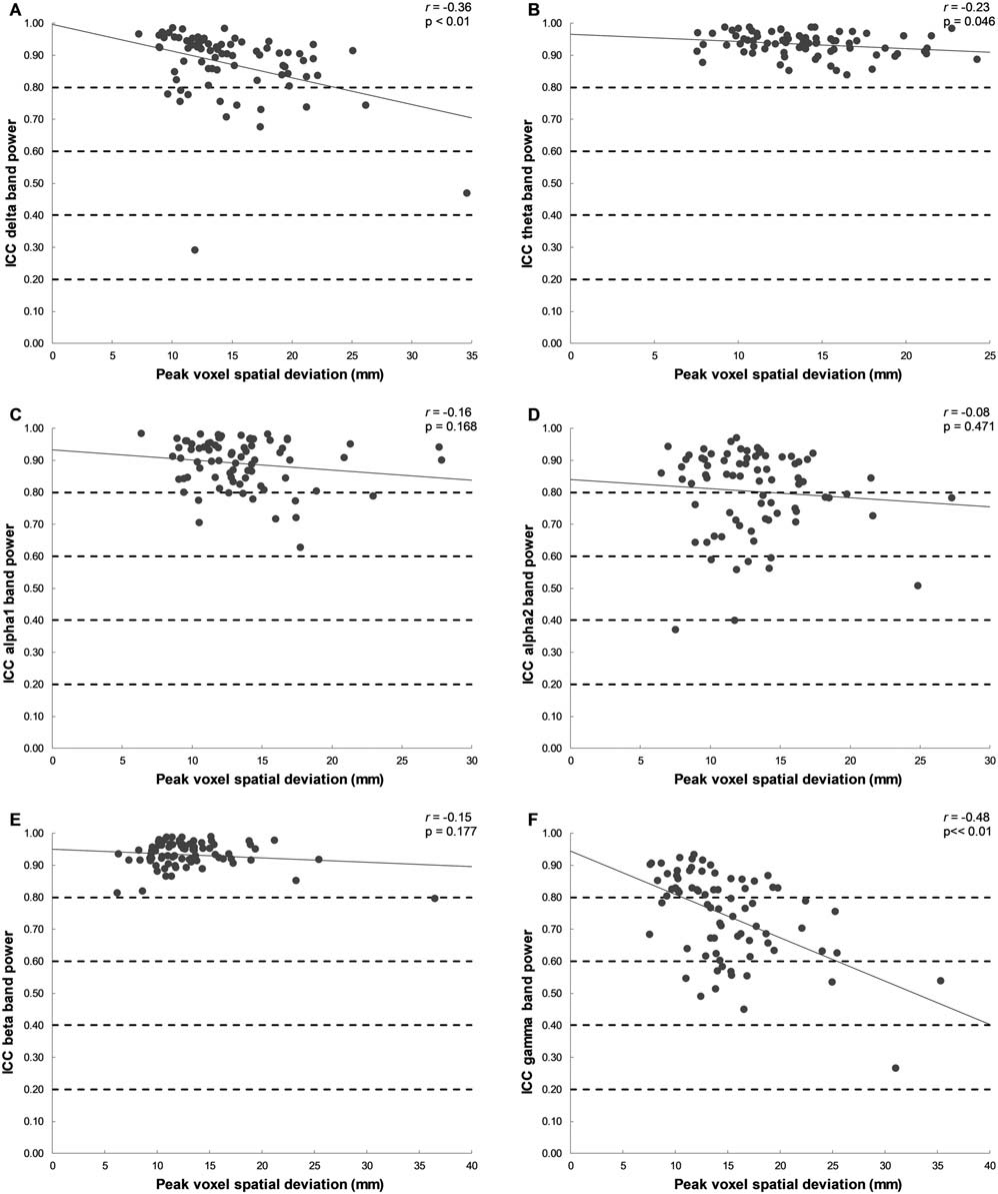
Consistency (ICC) of relative power averaged over subjects in relation to the distance between native versus template‐based peak voxel location. The ICC of relative power per ROI across template versus native MRI (averaged over subjects) is plotted (*y*‐axis) against the distance between the representative voxel (*x*‐axis) as obtained with the template versus native MRI (averaged over subjects), together with correlation (*r*) and *P* values (*P*).The dashed lines represent the rating of the ICCs: >0.80, very good; 0.61–0.80, good; 0.41–0.60, moderate; 0.21–0.40, fair; and <0.21, poor. Panels indicate frequency bands: A, delta band; B, theta band; C, lower alpha band; D, upper alpha band; E, beta band; F, gamma band.

We then explored Euclidean distance between peak voxels as chosen with the template versus native MRI approach as a correlate of the ICC of band‐limited relative power. Figure 3 shows that larger distances tended to relate to lower ICC, particularly in the delta, theta, and gamma bands. However, individual ICC values were still mostly classified as “good” or “very good,” even when distances were quite large (e.g., >2 cm). We therefore conclude that although spatial consistency of the selected representative voxels does have an effect on ICC values, consistency between the native versus MRI approach is largely maintained.

### Consistency of Regional and Global Power

**Figure 4:**
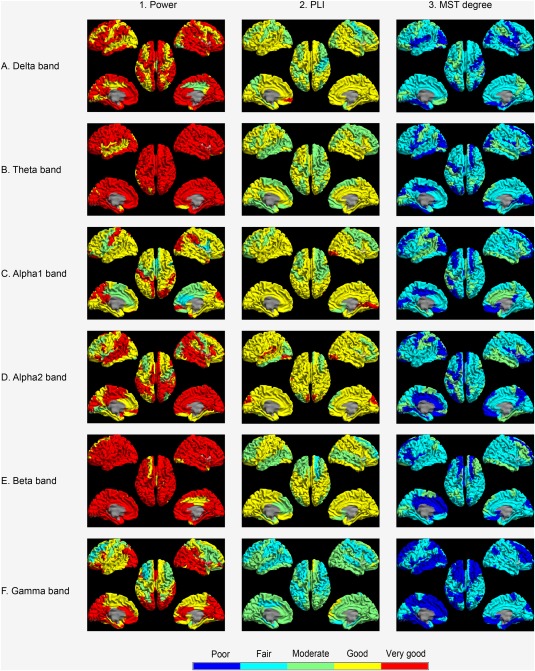
Consistency (ICC) of regional measures across epochs (*n* = 605) computed using template or native MRIs. Column 1 shows the intraclass correlation coefficient (ICC) for relative power per AAL region for all frequency bands as a color‐coded map. Note that overall good to very good ICCs were observed. Column 2 shows ICCs for the phase lag index (PLI) per region. Note that overall moderate to good ICCs were obtained. Column 3 indicates ICC per region for the MST degree. Note that overall fair ICCs were observed. Cold colors indicate poor and fair consistency, and hot colors indicate good and very good consistency.

**Figure 5:**
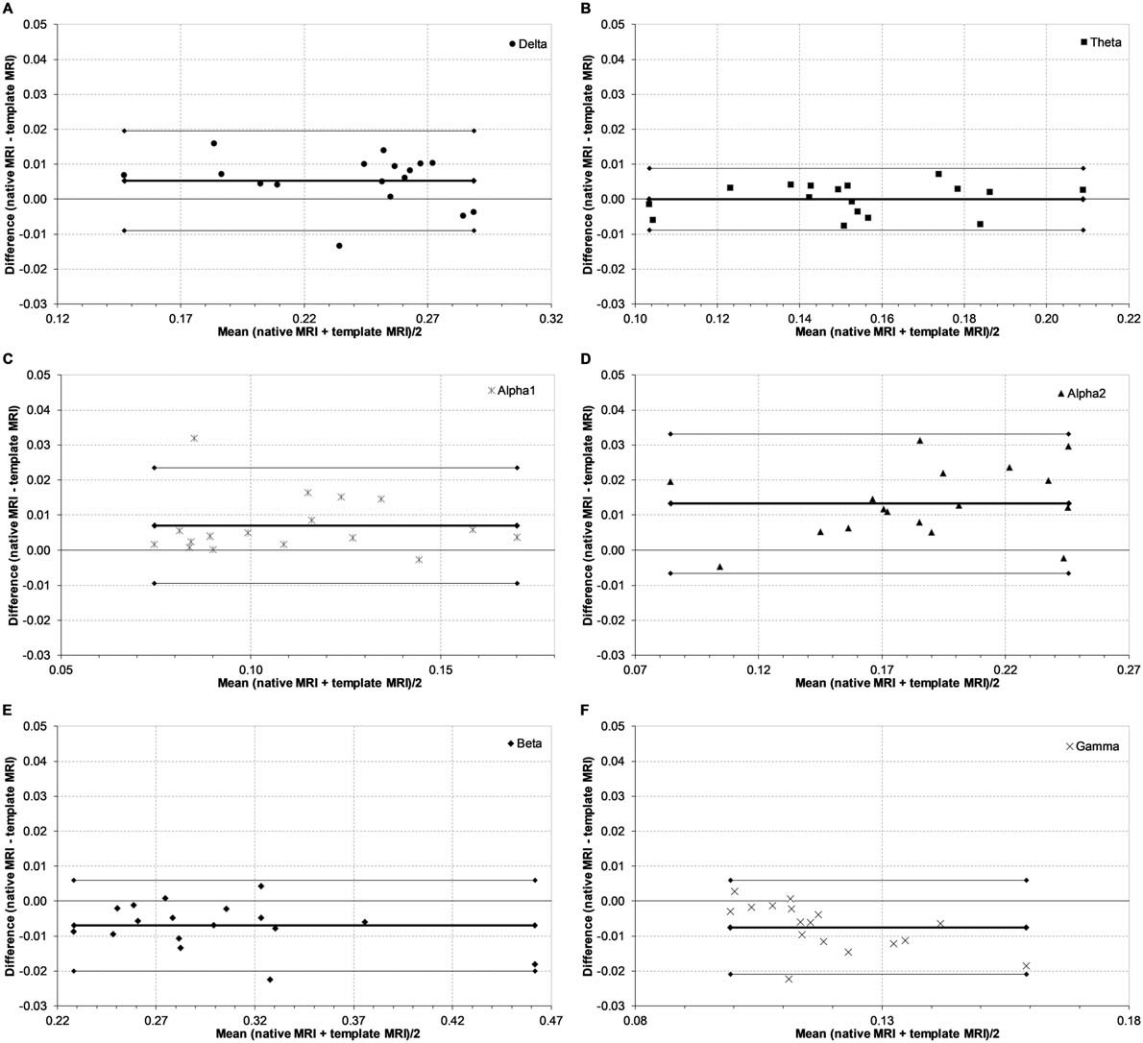
Bland–Altman plots of global relative power. Bland–Altman plots of the respective difference in global relative power across subjects (*n* = 17) between the template MRI and the native MRI for each individual against their respective means. Thin lines represent the limits of agreement corresponding to ±1.96 SD. The thick line represents the respective mean. Panels indicate frequency bands: A, delta band; B, theta band; C, lower alpha band; D, upper alpha band; E, beta band; F, gamma band.

Regional power per epoch (*n* = 605) was consistent across all 78 ROIs in the AAL atlas, as analyzed for each frequency band separately, showing overall good to very good ICCs (see Table 1 for peak voxel results, and Supporting Information, Appendix 1 for centroid results). We illustrate the consistency of relative regional power (and PLI and MST degree, see following section) across all regions and frequency bands in Figure 4 for the peak voxel method. Moreover, global relative power showed very good subject‐averaged ICCs per frequency band (Table 1). Figure 5 shows the Bland–Altman plots of relative power per frequency band at the subject level, and implicates no systematic differences between the two approaches.

### Consistency of Regional Connectivity and Network Metrics

The consistency of regional PLI across all epochs and across all 78 ROIs are illustrated in Figure 4, and show good to very good results for each frequency band (Supporting Information, Appendix 2). Furthermore, moderate to good results were obtained for the PLI in the delta to beta band, while lower consistency was found in the gamma band (Fig. 4 and Supporting Information, Appendix 3). The regional MST degree consistency across approaches was lower overall, with poor to moderate consistencies per frequency band and region (Fig. 4 and Supporting Information, Appendix 3).

### Consistency of Global Connectivity and Network Metrics

**Figure 6:**
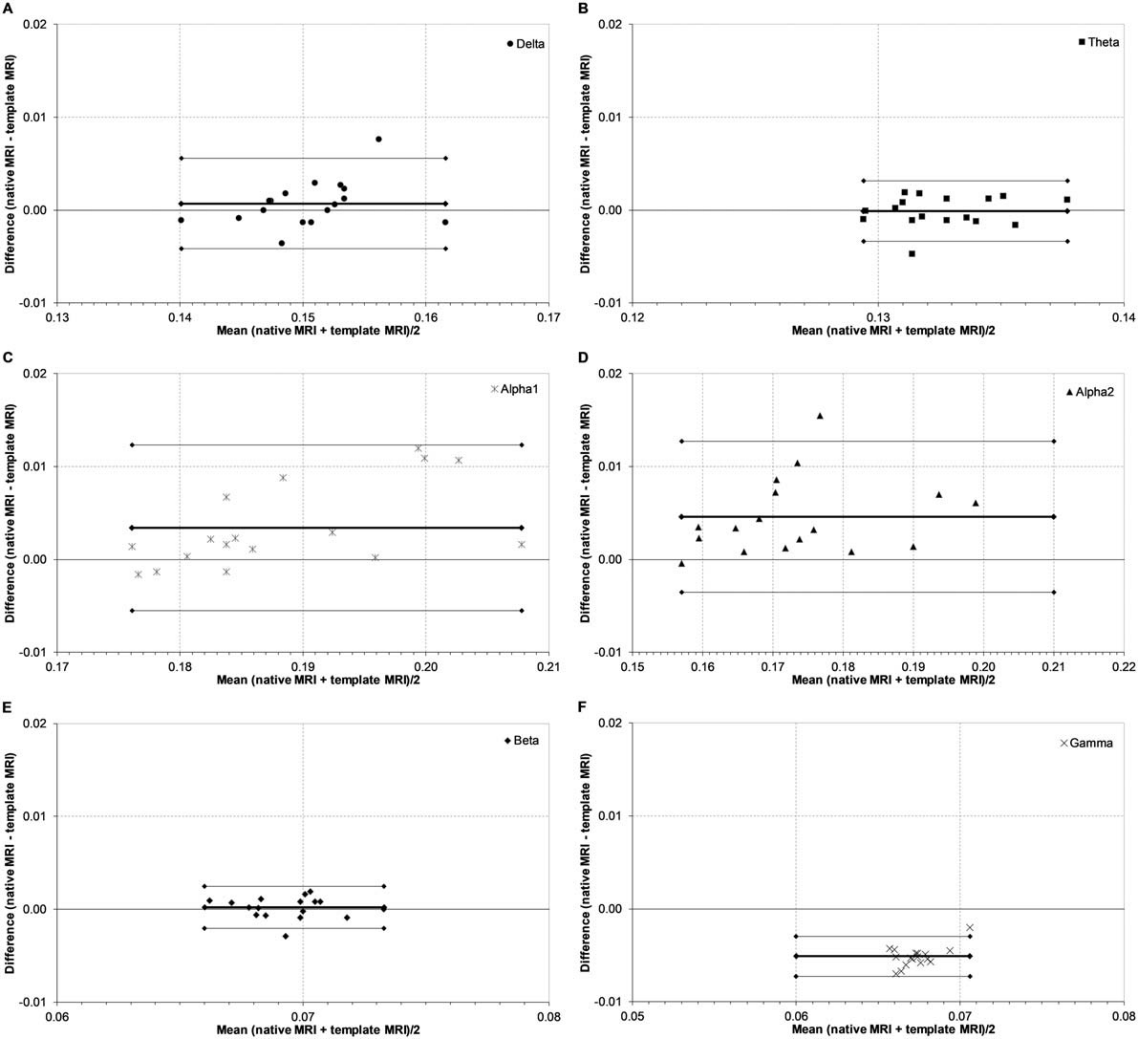
Bland–Altman plots of phase lag index. Bland–Altman plots of the respective difference in phase lag index across subjects (*n* = 17) between the template MRI and the native MRI for each individual against their respective means. Thin lines represent the limits of agreement corresponding to ±1.96 SD. The thick line represents the respective mean. Panels indicate frequency bands: A, delta band; B, theta band; C, lower alpha band; D, upper alpha band; E, beta band; F, gamma band.

We then investigated whether the global connectivity and network measures determined per subject (*n* = 17) were consistent when using a template versus native MRI (Table 1). For global PLI, ICCs were very good for most frequency bands (≥0.870), except for the gamma band. The Bland–Altman plots for the PLI indicated good limits of agreement between the template MRI and the native MRI (Fig. 6), as only one data point fell outside the 95% limits of agreement interval, as could be expected based on the 95% limit. Interestingly, gamma band global connectivity ICCs based upon the centroid approach, relative to the peak voxel approach predominantly described in this results section, were quite good (Table 1 and Supporting Information, Appendix 1).

Weighted clustering and path length were marked as having good (0.733) or very good (≥0.849) ICCs in the delta to beta bands, while the ICC values in the gamma band were marked as poor (Table 1).

**Figure 7:**
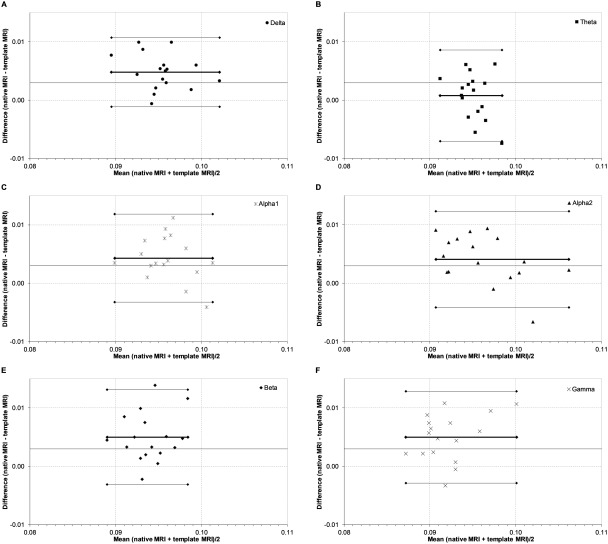
Bland–Altman plots for modularity. Bland–Altman plots of the respective difference in modularity across subjects (*n* = 17) between the template MRI and the native MRI for each individual against their respective means. Thin lines represent the limits of agreement corresponding to ±1.96 SD. The thick line represents the respective mean. Panels indicate frequency bands: A, delta band; B, theta band; C, lower alpha band; D, upper alpha band; E, beta band; F, gamma band.

Consistency of the number of modules was rated as moderate to good in the delta to beta bands (≥0.551) and was fair in the gamma band (0.320) (Table 1). The consistency of modularity Q was good in the delta band and upper alpha band, moderate in the lower alpha band and gamma band, fair in the beta band, and unreliable in the theta band. However, the Bland–Altman plot for the modularity still showed good limits of agreement (Fig. 7 and Table 1).

When determining consistency between template and native MRI for global MST measures, it can be noticed that the ICC of leaf fraction was rated as good across all frequency bands (Table 1). Furthermore, the ICC of MST diameter fluctuated between very good (delta, theta band), fair (lower alpha band), moderate (upper alpha, beta band), and good (gamma band) (Table 1). Bland–Altman plots for all global measures indicated acceptable to good limits of agreement, while no systematic differences were observed (figures not shown).

## DISCUSSION

The use of a native MRI for co‐registration is the current standard in MEG analysis, yet obtaining individual MRI may not always be feasible. By using a template MRI, the co‐registration and head model changes, which may affect beamformer‐reconstructed time series, and subsequent connectivity and network analyses. Therefore, the aim of this study was to examine the consistency of functional connectivity and network topology estimated from MEG data, using either a template or native MRI. Specifically, we examined what the possible inconsistencies of the template approach versus a native MRI would be for basic measures such as band‐limited relative power, but particularly for higher order measures such as functional connectivity and network topology. Our results showed overall good consistency of the use of a template MRI versus a native MRI for MEG co‐registration and an atlas‐based beamforming approach, particularly for relative power, functional connectivity and both regional and global network topology. Furthermore, consistency was maintained across two different atlas‐based beamforming approaches, with the peak voxel approach showing slightly less consistency as compared to the centroid approach.

With the centroid method, the location of the voxel that is chosen as representative for the ROI is solely based on geometry, thus the location of this voxel will only deviate if an anatomical mismatch is present between the template and native MRI. In contrast, with the peak voxel approach, the location of the representative voxel, that is, the peak voxel, is defined by the beamformer output. This location is not only directly affected by the aforementioned anatomical mismatch between template and native MRI, but also indirectly by the effect of this mismatch on the beamformer itself. We have shown that the spatial consistency of the virtual electrode locations, and therefore the expected consistency of subsequent outcome measures between the template and native MRI approach is indeed somewhat more stable for the centroid method than for the peak voxel method (Fig. 2). Furthermore, for all frequency bands, these distances correlated negatively with the ICC for relative power (although only significantly for the delta, theta, and gamma band), indicating that, as expected, larger spatial deviations generally relate to poorer consistency across approaches. However, even in the extreme case of a spatial deviation of 35 mm (Fig. 3, delta band), the ICC of the relative power for this particular region was still categorized as “moderate.” We therefore conclude that the use of a template versus native MRI leads to differences in the voxel selection, but that these differences have a relatively small impact on the consistency of the results of subsequent analyses.

When further investigating the consistency of outcome measures, we first showed that the time series themselves are highly consistent between the template and native MRI approach. Even time series from regions with the lowest consistency in peak voxel location, such as Heschl's gyrus, were moderately consistent across methods. These results therefore provide support for the idea that time series obtained with the template approach can be used as input for subsequent analyses.

The lowest consistency for global parameters averaged across subjects was observed for the MST leaf fraction and diameter, although these still yielded moderate to very good consistency in this relatively small sample. Owing to the higher order nature of these network measures, we expected their consistency to decrease nonlinearly: a basic, robust measure such as relative power will only be slightly affected by reconstruction errors [Beg et al., 2009; López et al., 2014; Steinstraeter et al., 2009]. However, these inconsistencies could be augmented in the next step of determining phase correlations. When taking the analysis one step further and calculating network topology, these inconsistencies could accumulate, potentially in a nonlinear manner (although, importantly [Fraschini et al., 2016] recently showed that MSTs can be accurately reconstructed even when PLI estimates are noisy). However, our results show that these higher level graph measures show larger but still acceptable inconsistencies across global measures. When looking at these complex measures on a more detailed scale, that is, at MST degree of individual regions, the consistencies decreased further (poor to moderate), and varied over regions. One reason for this variation could be that the location of the representative voxel was “incorrect” for some regions. This is most likely to be an issue for larger ROIs, where the distance between the selected location of the representative voxel and the correct location can become large, in combination with a high SNR, that is, when the spatial resolution of the beamformer is high so that activity is rapidly suppressed when moving away from the correct location [Barnes et al., 2004]. Importantly though, we have shown here that despite such potential errors at the local level, the patterns of functional connectivity and network topology are consistent at the global level.

The relatively weak results in the gamma band at regional and global levels might be attributed to a range of issues, which include the possibly larger distance between peak voxels as determined with the template versus native MRI approach (Figs. 2 and 3), muscle artifacts, and/or overall lower signal‐to‐noise ratio. More consistent results were obtained when using the centroid approach as opposed to the peak voxel method. Hypothetically, signals in the gamma frequency—with their possible overlap with artifacts and thus outliers in signal intensities—may be more sensitive to registration errors when focusing on the peak voxel, whereas choosing the centroid voxel may limit the influence of such artifacts. In general, though, the results for the peak voxel and centroid approaches were comparable, which is in line with previous research [Hillebrand et al., 2016; Zobay et al., 2015]. Furthermore, it is known that high‐frequency neural activity overlaps with the spectral bandwidth of muscle activity [Muthukumaraswamy, 2013]. Finally, the lower signal‐to‐noise ratio of gamma activity may limit the spatial resolution of the beamformer regardless of co‐registration method [Hillebrand and Barnes, 2003, 2011].

Vitamin E capsules were used to define anatomical matching between MEG and MRI coordinates. However, placing vitamin E capsules at anatomical landmarks may lead to larger co‐registration errors than for instance co‐registration with surface matching [Adjamian et al., 2004]. As a result, one might argue that differences between template and native MRI co‐registrations may have been imperfectly assessed, which may have led to higher consistency between the two approaches in comparison to the use of surface matching or head‐casts as a co‐registration method [Liuzzi et al., 2016; Troebinger et al., 2014]. Moreover, in the current study we did not use digitized head shapes to warp the template MRI to the subject's anatomy. Such an approach could potentially also improve the accuracy of the forward model and the reconstructed functional connectivity and networks, although our results suggest that such improvements would be modest, as ICCs were already moderate to very good. In case digitized head shapes are not available, co‐registration with a template MRI could be performed using fiducial markers. Such a co‐registration approach is less accurate than a surface matching approach [Adjamian et al., 2004], yet future studies should determine how much these extra errors add to connectivity and network reconstruction errors when using a template‐based approach.

Here, we considered the native MRI as the standard of analysis in MEG co‐registration. Our rationale was that if the assumptions behind a particular source reconstruction approach are valid [Hillebrand et al., 2005], then the accuracy of the inversion depends on the accuracy of the forward solution, and therefore on the accuracy of the co‐registration and MRI‐derived head model. Intuitively, the native MRI provides the most accurate information for the forward solution, and therefore the most accurate source reconstructions. However, in the presence of other errors, such as inaccurate source models, there could, perhaps counterintuitively, be situations where it is beneficial to be less accurate (either in terms of anatomy or signal‐to‐noise ratio) [Hillebrand and Barnes, 2003, 2011]. Moreover, it is possible that by using a template MRI individual variation in outcome measures is “suppressed.” This would make group‐effects more apparent and would therefore benefit group‐level analysis [Grabner et al., 2014; Huang et al., 2010]. Our results may support this notion, although only the use of model data may answer this question definitively. Our current goal was to investigate the consistency of experimental data analyzed with either a template or a native MRI.

The spatial resolution of MEG varies from millimeters to centimeters across the brain [Hillebrand and Barnes, 2002]. To perform our connectivity and network analyses, we opted to use the AAL atlas, although other atlases with higher resolutions are available [Evans et al., 2012]. This atlas roughly matches the spatial resolution of MEG data [Hillebrand and Barnes, 2002], and has as such been used many times by our group [Douw et al., 2013; Hillebrand et al., 2016; Olde Dubbelink et al., 2013; Tewarie et al., 2014b] and others [Dunkley et al., 2015; Hunt et al., 2016]. Moreover, the atlas‐based beamforming approach has been the subject of previous work [Hillebrand et al., 2012]. It has also been shown that the peak voxel method and the centroid‐based method give similar results [Hillebrand et al., 2016; Zobay et al., 2015]. Taken together, these results suggest that the resolution of the atlas that was used, namely 78 cortical ROIs in the AAL atlas, matches the resolution of the resting‐state MEG data. This conclusion is also supported by a recent study [Farahibozorg et al., 2017], in which different data‐driven parcellation approaches resulted in ∼70 ROIs, which is comparable to the number of ROIs used in our study.

We used the standard Montreal Neurological Institute's template, which is a widely used template and is based upon MR images of healthy participants, as a substitute for participants’ individually acquired MRI. Although the precision of the use of a template MRI could theoretically be improved by using population specific templates for different ages [Richards et al., 2016], head sizes, and head shapes, for the subsequent connectivity and network analysis [Fillmore et al., 2015; Rorden et al., 2012], the current results generally support its use in the general population. However, we note that the use of a template MRI might become problematic when patients suffer from brain atrophy or intracranial lesions, as these may yield much larger co‐registration issues than explored in the current study.

In this work, we used beamforming as an inverse solver for MEG data. It remains an open question whether our conclusion that a native MRI can be replaced by a template without introducing large errors in connectivity or network estimates generalizes to alternative source reconstruction approaches. In particular, inverse solvers that use hard constraints on, for example, the location and orientation of the cortical surface, such as minimum norm based approaches [Baillet et al., 2001], are likely to suffer from the inaccuracies in these constraints that may occur due to the use of a template MRI [Hillebrand and Barnes, 2003]. Similarly, we only tested the use of a template with MEG data. A recent study that examined the consistency of source localization and connectivity estimates in EEG, shows variability in connectivity and this variability was seen across inverse methods and toolboxes [Mahjoory et al., 2017]. For EEG measured with a limited number of electrodes, the spatial resolution is lower than that for MEG to begin with; hence, the effects of inaccuracies due to the use of a template MRI instead of a native MRI will probably be small. However, for EEG with hundreds of channels, the accuracy of the forward solution becomes more important, such that the use of a template could potentially have detrimental effects. Related to this issue, we have used a simple head model in this work. Owing to the use of a simple head model, relatively large localization errors were to be expected to begin with [Stenroos et al., 2014]. Again, using more sophisticated head models, such as Finite/Boundary Element Models (FEM/BEM) would require more accurate information from MRI, and we therefore expect the use of a template to have more detrimental effects with such head models. In general, the effect of replacing a native MRI with a template is difficult to predict for a particular situation, as it will depend on the details of each source (e.g., location, amplitude, orientation), and other circumstances (e.g., SNR, number of other active sources and their relative strengths and locations, type of head model used, MEG system used, inverse solver used, etc.). Although such details remain uncertain in the current study, we envisage that this would form a fruitful area of research for future simulation studies. Similarly, simulations could also further quantify how tolerant phase relationships are to reconstruction errors in source location and amplitude. Our results suggest that even in the presence of localization errors (Fig. 2) and errors in the reconstructed time series (Table 2), the estimated phase relationships remain rather consistent (Fig. 4).

Here, we have shown, using an experimental data set, what the impact is of using a template versus native MRI for co‐registration of MEG data on the global and regional properties of reconstructed functional networks. We observed that functional connectivity and functional networks can be reconstructed with reasonable consistency across approaches. This may encourage others to also apply sophisticated analyses to MEG datasets that have no native MRIs available, which they otherwise may not have considered.

## Supporting information

Supporting Information

Supporting Information

Supporting Information

Supporting Information
