# Supplementary material for: Consistency of magnetoencephalographic functional connectivity and network reconstruction using a template versus native MRI for co‐registration
Source: Hum Brain Mapp. 2017 Oct 8;39(1):104–19. doi: 10.1002/hbm.23827 (PMC5725722; doi:10.1002/hbm.23827)
Supplement: Supplementary file 1 — Supporting Information [file HBM-39-104-s001.docx]

Appendix 1. Global consistency between results obtained when using the template or native MRI approach (with the centroid method)

|  | Delta | | | Theta | | | Lower alpha | | |
| --- | --- | --- | --- | --- | --- | --- | --- | --- | --- |
|  | ICC | 95% CI | Rating | ICC | 95% CI | Rating | ICC | 95% CI | Rating |
| Power | 0.962 | 0.541 – 0.991 | VG | 0.987 | 0.963 – 0.995 | VG | 0.950 | 0.722 – 0.985 | VG |
| PLI | 0.899 | 0.727 – 0.963 | VG | 0.804 | 0.469 – 0.929 | VG | 0.791 | 0.443 – 0.923 | G |
| Cw | 0.936 | 0.820 – 0.977 | VG | 0.754 | 0.310 – 0.911 | G | 0.660 | 0.123 – 0.874 | G |
| Lw | 0.879 | 0.673 – 0.956 | VG | 0.795 | 0.445 – 0.925 | G | 0.802 | 0.469 – 0.928 | VG |
| Modularity | 0.143 | -0.087 – 0.497 | P | 0.684 | -0.140 – 0.901 | G | 0.826 | 0.114 – 0.950 | VG |
| Number of modules | 0.555 | -0.094 – 0.831 | M | 0.662 | -0.007 – 0.883 | G | 0.861 | 0.577 – 0.951 | VG |
| MST leaf fraction | 0.760 | 0.002 – 0.927 | G | 0.823 | 0.234 – 0.946 | VG | 0.776 | 0.227 – 0.925 | G |
| MST diameter | 0.707 | 0.205 – 0.893 | G | 0.797 | 0.459 – 0.926 | G | 0.711 | 0.208 – 0.895 | G |
|  | Upper alpha | | | Beta | | | Gamma | | |
|  | ICC | 95% CI | Rating | ICC | 95% CI | Rating | ICC | 95% CI | Rating |
| Power | 0.934 | 0.502 – 0.982 | VG | 0.973 | 0.439 – 0.994 | VG | 0.634 | -0.165 – 0.903 | G |
| PLI | 0.830 | 0.539 – 0.938 | VG | 0.839 | 0.561 – 0.941 | VG | 0.791 | 0.443 – 0.923 | G |
| Cw | 0.936 | 0.775 – 0.979 | VG | 0.826 | 0.404 – 0.942 | VG | 0.660 | 0.123 – 0.874 | G |
| Lw | 0.815 | 0.500 – 0.932 | VG | 0.825 | 0.531 – 0.936 | VG | 0.802 | 0.469 – 0.928 | VG |
| Modularity | 0.676 | 0.070 – 0.885 | G | 0.682 | -0.021 – 0.893 | G | 0.826 | 0.114 – 0.950 | VG |
| Number of modules | 0.630 | -0.024 – 0.867 | G | 0.646 | 0.001 – 0.874 | G | 0.861 | 0.577 – 0.951 | VG |
| MST leaf fraction | 0.795 | -0.104 – 0.944 | G | 0.741 | -0.149 – 0.926 | G | 0.776 | 0.227 – 0.925 | G |
| MST diameter | 0.664 | 0.132 – 0.875 | G | 0.369 | -0.265 – 0.740 | F | 0.711 | 0.208 – 0.895 | G |

N=17 ; df 16
ICC, intraclass correlation coefficient ; 95 CI, 95% confidence interval
VG= Very good ICC >.80; G= Good ICC .61 - .80; M= Moderate ICC .41 - .60; F= Fair ICC .21 - .40; P= Poor ICC <.21
*Not Reliable
