# Supplementary material for: Consistency of magnetoencephalographic functional connectivity and network reconstruction using a template versus native MRI for co‐registration
Source: Hum Brain Mapp. 2017 Oct 8;39(1):104–19. doi: 10.1002/hbm.23827 (PMC5725722; doi:10.1002/hbm.23827)
Supplement: Supplementary file 2 — Supporting Information [file HBM-39-104-s002.docx]

Appendix 2: Regional consistency between results obtained when using the template or native MRI approach for relative power averaged across all epochs (n=605) and all 78 ROIs per frequency band (with the peak voxel method)

| Power |  |  | Delta | Theta | Lower alpha | Upper alpha | Beta | Gamma |
| --- | --- | --- | --- | --- | --- | --- | --- | --- |
| ICC | <.21 | Poor |  |  |  |  |  |  |
|  | .21 - .40 | Fair |  |  | 3 |  |  | 1 |
|  | .41 - .60 | Moderate | 2 |  | 13 | 8 |  | 12 |
|  | .61 - .80 | Good | 19 | 8 | 51 | 32 | 5 | 32 |
|  | >.81 | Very Good | 57 | 70 | 11 | 38 | 73 | 33 |

ICC, intraclass correlation coefficient
