# Supplementary material for: Consistency of magnetoencephalographic functional connectivity and network reconstruction using a template versus native MRI for co‐registration
Source: Hum Brain Mapp. 2017 Oct 8;39(1):104–19. doi: 10.1002/hbm.23827 (PMC5725722; doi:10.1002/hbm.23827)
Supplement: Supplementary file 4 — Supporting Information [file HBM-39-104-s004.docx]

Appendix 4: Regional consistency between results obtained when using the template or native MRI approach for MST degree averaged across all epochs (n=605) and all 78 ROIs per frequency band (with the peak voxel method)

| MST degree |  |  | Delta | Theta | Lower alpha | Upper alpha | Beta | Gamma |
| --- | --- | --- | --- | --- | --- | --- | --- | --- |
| ICC | <.21 | Poor | 20 | 19 | 17 | 21 | 16 | 34 |
|  | .21 - .40 | Fair | 46 | 49 | 45 | 38 | 51 | 44 |
|  | .41 - .60 | Moderate | 12 | 10 | 16 | 19 | 11 |  |
|  | .61 - .80 | Good |  |  |  |  |  |  |
|  | >.81 | Very Good |  |  |  |  |  |  |

ICC, intraclass correlation coefficient
